# The Electrostatically Formed Nanowire: A Novel Platform for Gas-Sensing Applications

**DOI:** 10.3390/s17030471

**Published:** 2017-02-26

**Authors:** Gil Shalev

**Affiliations:** 1 Department of Electrical and Computer Engineering, Ben-Gurion University of the Negev, POB 653, Beer-Sheva 84105, Israel; glshalev@bgu.ac.il; 2 Ilse-Katz center for Nanotechnology, Ben-Gurion University of the Negev, POB 653, Beer-Sheva 84105, Israel

**Keywords:** electrostatically formed nanowire, gas sensor, ethanol, silicon, Kelvin probe force microscopy

## Abstract

The electrostatically formed nanowire (EFN) gas sensor is based on a multiple-gate field-effect transistor with a conducting nanowire, which is not defined physically; rather, the nanowire is defined electrostatically post-fabrication, by using appropriate biasing of the different surrounding gates. The EFN is fabricated by using standard silicon processing technologies with relaxed design rules and, thereby, supports the realization of a low-cost and robust gas sensor, suitable for mass production. Although the smallest lithographic definition is higher than half a micrometer, appropriate tuning of the biasing of the gates concludes a conducting channel with a tunable diameter, which can transform the conducting channel into a nanowire with a diameter smaller than 20 nm. The tunable size and shape of the nanowire elicits tunable sensing parameters, such as sensitivity, limit of detection, and dynamic range, such that a single EFN gas sensor can perform with high sensitivity and a broad dynamic range by merely changing the biasing configuration. The current work reviews the design of the EFN gas sensor, its fabrication considerations and process flow, means of electrical characterization, and preliminary sensing performance at room temperature, underlying the unique and advantageous tunable capability of the device.

## 1. Introduction

The advancement in nanotechnology has elicited the development of nanosensors, which can detect the presence of nanoparticles and molecules or monitor various physical quantities (pressure, temperature, etc.) on a nanometer scale. Potentially, such nanosensors can very efficiently couple to nanoscale interactions due to their nanoscale dimensions. Nanosensors are very promising candidates for medical diagnostic applications and for health monitoring and management [1,2,3,4,5]. A continuous effort is underway for improving the stability, robustness, response time, and reproducibility of nanosensors [5,6,7,8]. 

One-dimensional (1D) chemical sensors that comprise nanowires or nanotubes of various materials exhibit an ultrasensitive sensing performance [9,10,11,12,13,14,15,16,17,18,19,20]. Carbon nanotubes (CNTs), in particular, demonstrate outstanding potential for gas-sensing applications, which have been rapidly developed over the past few years [16,17,18,19,20]. Ding et al. demonstrated the sensing of acetone vapors with single-wall CNTs (SWNTs), with a limit of detection (LOD) of a few parts per million (ppm) [19]. Chen et al. demonstrated the sensing of nitric oxide (NO) in concentrations as low as 590 parts per quadrillion (ppq) at room temperature [16], and Kumar et al. recently reported an LOD of 125 parts per trillion (ppt) for NO_2_ with an SWNT sensor [21]. However, the reproducibility of CNTs with consistent properties and with performance ability that is required for sensing applications is still challenging [22]. The metal oxide sensor (MOS) is probably the most popular, and also commercially available, sensing technology. The MOS measures the change in the electrical resistance of the device upon an interaction with the target analyte. Recently, Cho et al. demonstrated a patterned p-type polycrystalline MOS nanowire array with an improved sensitivity and response time toward various volatile organic compounds (VOCs) [23], and Katwal et al. fabricated a novel zinc oxide nanowire–nanotube hybrid-based sensor with a good response to specific VOCs that serve as markers for breast cancer [24]. Similarly, various other studies demonstrated the outstanding sensing potential of MOS technology for VOC detection [25,26,27]. Still, the MOS technology operates at elevated temperatures that lead to high power consumption and complicated device assembly. Gas sensors based on silicon nanowire (SiNW) field-effect transistors demonstrate outstanding LODs. SiNW transistors are similar to conventional metal-oxide-semiconductor (MOS) transistors, but the top metal gate is replaced with a molecular gate that is, in effect, the target molecules. Once target molecules that carry a net electric charge or a net electric dipole are adsorbed upon the top active oxide surface (or the chemically modified oxide surface) of the SiNW field-effect transistor, the charge or dipole induces respective electric fields, which act upon the conducting channel. For example, negatively charged molecules induce electric fields that deplete an electron-conducting channel, and a decrease in current is recorded. McAlpine et al. demonstrated the sensing of NO_2_ in a concentration range of parts per billion (ppb) [9] with unmodified silicon nanowire arrays on flexible plastic substrates, and Lichtenstein et al. demonstrated the detection of explosives in liquid phase in a concentration range of ppq [14] with chemically modified silicon nanowire arrays. Shehada et al. reported ultrasensitive, molecularly modified silicon nanowire transistors for sensing VOCs, which were used to diagnose gastric cancer. Silicon nanowires are fabricated by using either bottom-up or top-down techniques. The most popular bottom-up technique is the vapor–liquid–solid (VLS) growth technology, which was demonstrated to produce nanowires suitable for gas sensing [11,12,13]. However, VLS growth is inherently unsuitable for mass production, as it does not support a deterministic design and because the fabrication of VLS nanowires requires gold catalysts and, therefore, the nanowires suffer from gold contamination that degrades their electrical performance [28]. Conventional top-down nanowire fabrication techniques, on the other hand, entail considerable expenditure due to the high-end lithography required to reach the nanometer scale. Therefore, the top-down approach is not feasible for most gas-sensing applications, which must be cost-effective. 

Taken together, sensors composed of nanomaterials and sensors that are of a nanometric size still face great challenges in terms of their suitability for mass production, the robustness of their fabrication processes, device and performance, their reproducibility, and so forth. Therefore, the commercialization of products based on nanosensors is yet to be developed and demonstrated. A new paradigm is thus required, which can provide the sensing capabilities associated with nanoscale sensors while overcoming the myriad obstacles associated with the realization of nano-based products.

The current work reviews the electrostatically formed nanowire (EFN) gas sensor, which was first introduced as a specific, real-time, and label-free biosensor for the detection of protein–protein interactions at femtomolar concentrations [29]. The advantages of the EFN gas sensor over other gas sensors are twofold. First, the EFN sensor is fabricated by using conventional silicon fabrication techniques with relaxed design rules, resulting in low cost, robustness, and suitability for mass production. Second, the nanowire in an EFN device is not physically defined but, rather, electrostatically defined, and, therefore, the dimensions, shape, and even the location of the nanowire are defined post-fabrication. The below reviews the EFN sensor and discusses design considerations, fabrication technology, and sensing performance in terms of LOD, tunable sensitivity, and tunable dynamic detection range.

## 2. Design Considerations of EFN Devices

**Figure 1:**
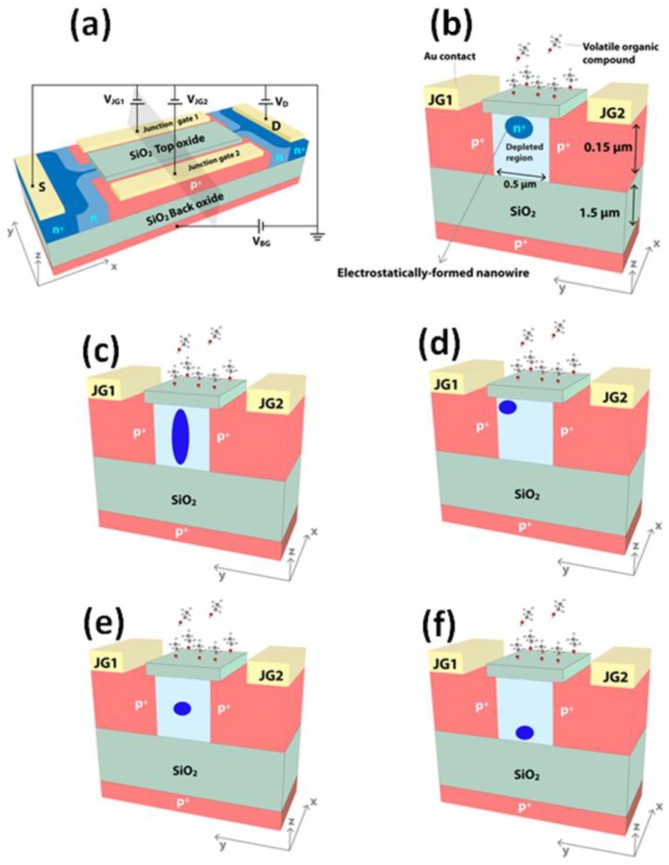
Design and possible modes of operation of an electrostatically formed nanowire (EFN) gas sensor. (**a**) EFN gas sensor with an electrical biasing configuration; (**b**) small EFN at the top of the bulk silicon-on-insulator (SOI): V_JG1_ = V_JG2_ < 0 V and V_BG_ < 0 V; (**c**) elongated EFN: V_JG1_ = V_JG2_ < 0 V and V_BG_ = 0 V; (**d**) asymmetric biasing—small EFN close to JG1: V_JG1_ = 0 V, V_JG2_ < 0 V, and V_BG_ < 0 V; (**e**) V_JG1_ = V_JG2_ < 0 V and V_BG_ = 0 V. Gradient of donor concentration peaking at the center of the SOI is required to have the EFN in the middle of the bulk SOI; (**f**) V_JG1_ = V_JG2_ < 0 V and V_BG_ = 0 V. Gradient of donor concentration peaking at the bottom of the SOI is required to have the EFN in the bottom of the bulk SOI. (**a**) and (**b**) are reprinted from [33], with permission of Tsinghua University Press and Springer-Verlag Berlin Heidelberg, 2015.

The EFN gas sensor is based on a silicon-on-insulator (SOI) technology, as illustrated schematically in Figure 1a. The EFN gas sensor is an accumulation-type transistor with a source-to-drain conducting channel controlled by four surrounding gates: an MOS backgate (V_BG_); two lateral gates comprising PN junctions (V_JG1_ and V_JG2_) in a back-to-back configuration, each located on either side of the conducting channel; and a top field-effect gate, where the strength and extent of the induced field is determined by the electric charge or dipole of the target molecule. The EFN gas sensor resembles the G^4^ field-effect transistor, which was developed in 2002 [30,31,32], but the top metal gate is replaced by the top molecular gate. Below, we describe an n-type accumulation-type EFN device. Negative bias of the backgate depletes the SOI from majority carriers (electrons) and confines the accumulated channel to the top of the SOI. Hence, the backgate determines the “height” of the channel in the bulk SOI. A negative bias of the V_JG_s forces the PN junctions to operate in reverse mode and, therefore, increases the depletion regions on both sides of the metallurgical junctions. Accordingly, the higher the reverse biasing, the larger are the depletion regions and the narrower is the conducting channel. In that sense, the junction gates control the width of the channel in the bulk SOI, where sufficient biasing induces a transition from a wide conducting channel into an EFN. Similarly, the lateral location of the conducting channel (or EFN) can be controlled through an asymmetrical biasing of the V_JG_s (i.e., V_JG1_ ≠ V_JG2_). However, reverse biasing of the PN junctions is limited by the junction breakdown voltage, which is determined by the dopant concentration (donors) of the bulk SOI. To use a higher reverse bias, the dopant concentration in the bulk SOI needs to be smaller; however, a smaller dopant concentration can result in the absence of carriers for the formation of the channel. We previously demonstrated an EFN gas sensor, in which the distance between the lateral metallurgical junctions was ~650 nm (365 nm i-line lithography), to show that correct biasing of V_BG_ and V_JG_s generates EFN diameters as small as 16 nm [33]. The top chemical gate is, in effect, the electronic signal generated by the target molecule; the electric charge or electric dipole of the target molecule induces an electric field that generates perturbation in the underlying EFN. For example, for an n-type SOI, a positively/negatively charged target molecule will generate a local accumulation/depletion in the underlying EFN, and, therefore, an increase/decrease in the source-drain current (I_DS_). Figure 1b–f demonstrates how the location, shape, and size of the conducting channel are manipulated in an EFN gas sensor. Figure 1b shows how the channel is shaped into an EFN, which is located at the top of the bulk SOI; the lateral dimensions of the EFN are determined by V_JG_s and the location along the z-axis is determined by V_BG_. The roundness of the EFN is due to the overlap between the depletion region due to V_BG_ and the depletion regions due to V_JG_s. The vertical location of the EFN is of importance for sensing applications. On the one hand, the EFN is close to the active sensing area when it is located at the top of the bulk SOI, which, in principal, improves both LOD and sensitivity. On the other hand, the interface of the SOI and the top SiO_2_ active sensing layer introduces fluctuations in mobility and/or in carrier density [34]. Therefore, an EFN adjacent to this interface will suffer from high levels of electronic 1/f noise, which impairs LOD and sensitivity. The overall sensing performance of an EFN gas sensor with respect to the above two contradicting effects (proximity vs noise) is yet to be studied. Figure 1c shows how the conducting channel is shaped into an elongated EFN. This configuration is realized for a negative V_JG_ to narrow the channel and V_BG_ = 0 V. In Figure 1d, the EFN is located at the side of the bulk SOI, using V_JG1_ ≠ V_JG2_ and V_BG_ ≠ 0 V, and in Figure 1e,f, the EFN is located at the middle and bottom (respectively) of the bulk SOI, using a gradient of dopant concentration, such that the concentrations are maximized in the middle and the bottom of the bulk SOI, respectively. In the following review, V_JG1_ = V_JG2_ = V_JG_ is assumed, unless otherwise noted.

The EFN is inherently different from both top-down and bottom-up conventional SiNWs. A SiNW is a three-dimensional structure, in which the electronic potential confinement is chemical due to the discontinuity in material properties at the interface between the SiNW and the cladding/passivation layer (for example, air or SiO_2_). In the EFN gas sensor, by contrast, the confinement is fully electrostatic (parabolic potential) due to the nature of the external applied bias. This acute difference in confinement potential between physical SiNWs and EFN entails implications on the performance of gas sensing, but these are yet to be determined. The EFN offers distinct advantages over SiNWs. For example, performance degradation due to inhomogeneous dopant distribution and metal catalysts in bottom-up SiNW does not occur in EFN gas sensors, as the EFN exists inside a single crystalline silicon, which entails high bulk mobility, high signal-to-noise ratio, crystalline purity, and homogeneous dopant distribution. In addition, in contrast with bottom-up or top-down SiNWs, EFN gas sensors are fabricated by using standard silicon fabrication techniques, which enable mass production, low cost, robustness, and high electronic performance. Finally, EFN is an electrostatically shaped post-fabrication, such that only a single design of an EFN gas sensor needs to be fabricated. The shape, location, and size of the EFN are determined post-fabrication and can be tailored to the sensing specificities of a given target molecule and application. 

## 3. Fabrication of EFN Devices

**Figure 2:**
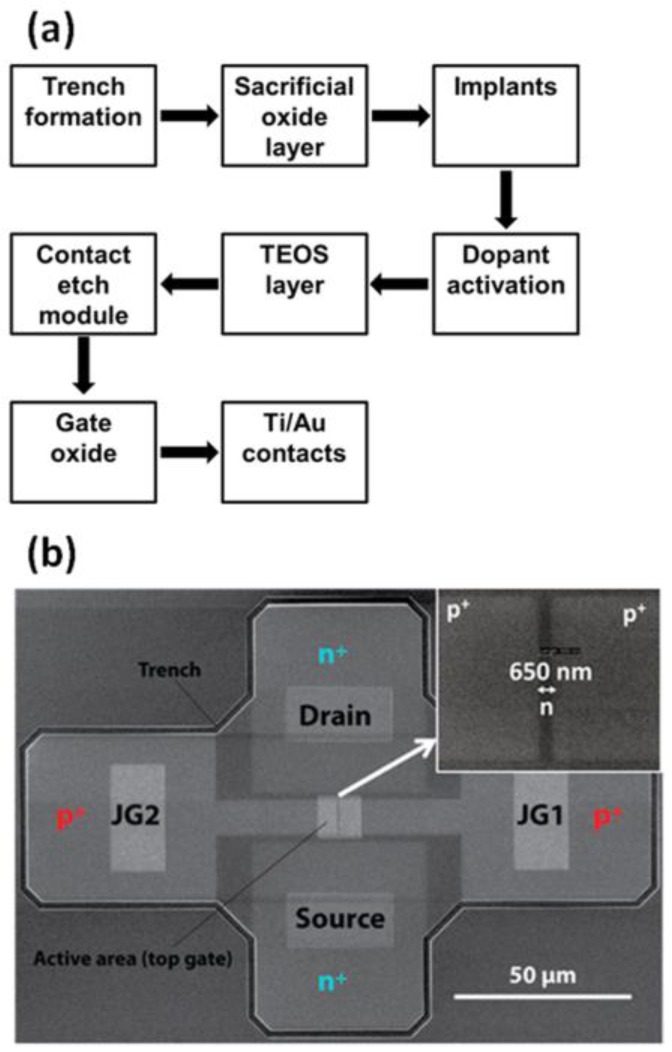
(**a**) Schematic fabrication process flow of an EFN gas sensor; (**b**) scanning electron microscopy image of an EFN device. Note that the critical dimension (650 nm) is the distance between the two metallurgical PN junctions on both sides of the conducting channel. (**b**) is reprinted with permission from [35]. Copyright 2016 American Chemical Society.

Figure 2a describes the fabrication of n-type EFN gas sensors. P-type (boron) SOI wafers are the starting material, with an SOI thickness of ~150 nm and a buried oxide (BOX) thickness of 1 µm. The first fabrication module is the formation of the trench that electrically isolates each EFN gas sensor from its surroundings. The trench module starts with trench lithography and a subsequent dry-etch process, which opens the SOI all the way down to the underlying BOX, followed by removal of the resist. Then, a thermal treatment is employed to form a thin (~100 nm) sacrificial oxide layer to be used in the subsequent implant steps. The first implant step is an arsenic blanket implant (i.e., without lithographic definition) to set the dopant concentration of the bulk SOI. This step is pivotal to the performance of the EFN gas sensor, as a gradient in dopant concentration can be introduced to determine the location of the conducting channel at a zero bias. Therefore, implant simulations are conducted to determine implant dose and energy. Time-of-flight secondary ion mass spectroscopy (TOF-SIMS) is performed to determine the actual implant profile in the bulk SOI. Next, the source and drain regions are formed. Lithography is used to define source/drain areas, succeeded by arsenic implantation to provide highly doped source and drain. The lithography and implant steps are repeated for the junction gates, using boron. The junction gate lithography defines the critical dimension (CD) of the process, as the distance between the two junction gates is by far the smallest detail in the EFN fabrication process. The implant steps are succeeded by thermal annealing treatments for dopant activation. A tetraethoxysilane (TEOS) layer of about 40 nm is deposited to passivate and isolate the active silicon areas from the metal wiring connecting the devices to the outside world. The contact areas are now defined by using lithography followed by a wet etch with hydrofluoric (HF) acid all the way down to the SOI. Next, a thermal oxide is grown (diffusion furnace), which will serve as a high-quality active sensing area. Finally, the Ti/Au contacts are formed by using the lift-off technique. 

Figure 2b presents a scanning electron microscope (SEM) image of an EFN gas sensor before the formation of the Ti/Au contacts; the inset shows the CD of the process, which, in this specific device, was set to 650 nm. The effective length of the conducting channel is 10 µm. The trench that binds and isolates the device is noticeable, as well as the active sensing area, which was formed by using wet etch, together with the source/drain and junction gates contacts. The doped p-type and n-type areas are also evident. 

## 4. Electrical Performance and Characterization of EFN Devices

**Figure 3:**
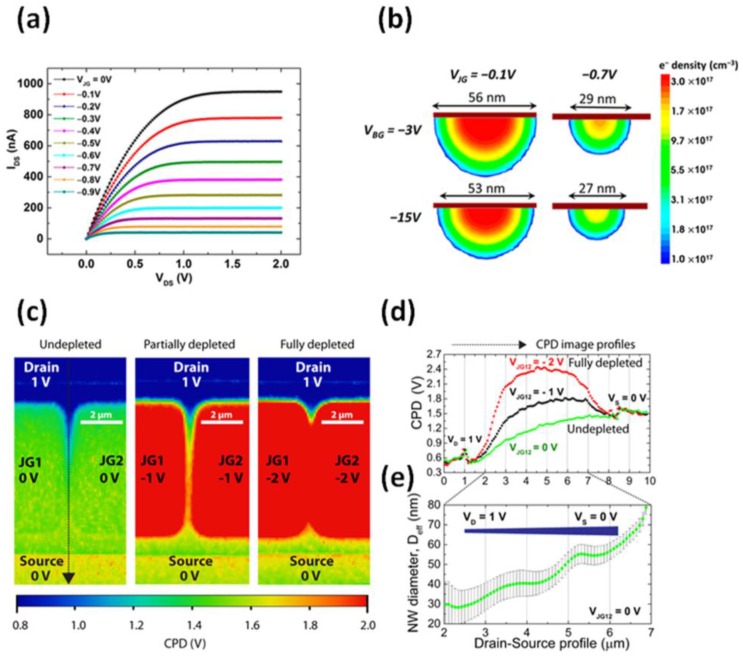
Electrical characterization of an EFN gas sensor. (**a**) An I_DS_ vs V_DS_ curve for various V_JG_ values. Note that I_DS_ decreases as V_JG_ becomes more negative; (**b**) numerical simulations of the electron density in the conducting channel of an EFN gas sensor; (**c**) contact potential difference (CPD) images of the EFN active area; and (**d**) CPD profiles along the EFN channel from source to drain, for three different cases: undepleted, partially depleted, and fully depleted. V_BG_ was maintained at 0 V; (**e**) the EFN diameter in the axial direction along the nanowire was extracted from the measured CPD profile at V_JG12_ = 0 V. In both (**d**) and (**e**), V_JG12_ = V_JG_. (**a**,**b**) are reprinted with permission from [35]. Copyright 2016 American Chemical Society. (**c**–**e**) are reprinted from [33], with permission of Tsinghua University Press and Springer-Verlag Berlin Heidelberg 2015.

The transistor characteristics (I_DS_ vs drain-source voltage, V_DS_) of an n-type EFN device are shown in Figure 3a for a range of junction gate voltages [35]. It is easy to see that I_DS_ decreases as V_JG_ becomes more negative. A negative V_JG_ forces the PN junctions on both sides of the channel into a reverse-biasing mode, which increases the depletion areas on both sides of the conducting channel and, therefore, narrows the channel, resulting in a transition from a wide conducting channel to an EFN. This decrease in channel diameter is clearly evident when solving the device numerically; the electrical performance of an EFN device was calculated with a three-dimensional device simulator (Synopsys TCAD Sentaurus, MountainView, CA, USA), which solves the Poisson and the continuity equations. A cross-section of the simulated electron density distribution at half length of the channel between the drain and source is shown in Figure 3b. We define the effective diameter of the EFN (D_eff_) as the full width at half-maximum (FWHM) of the electron density. Importantly, in the present case, the lithographic distance between the junction gates is 650 nm (CD). Remarkably, even at V_JG_ = −0.1 V and V_BG_ = −3 V, D_eff_ of the conducting channel is reduced to 56 nm and the channel is transformed into an EFN. The decrease in channel diameter from 650 nm to below 100 nm is attributed to the built-in potentials at the junctions, which ensure the formation of an EFN even at a zero bias. Applying even higher negative biases at the junction gates and backgate (−0.7 V and −15 V, respectively) conclude an EFN with D_eff_ = 27 nm. Generally, the simulations clearly indicate that the junction gates control the width of the channel and the backgate controls the “height” of the channel. However, to some extent, the junction gates affect the height of the channel and the backgate affects the width of the channel, as parts of the depletion regions of the junction gates and of the backgate overlap.

Kelvin probe force microscopy (KPFM) was used to experimentally determine the diameter of the EFN. KPFM measures the contact potential difference (CPD) between a sample and the KPFM tip with a millivolt sensitivity and a spatial resolution in the nanometer range [36]. Thus, the CPD allows the determination of the Fermi energy of the sample. Figure 3c presents CPD x–y maps of an EFN gas sensor under three biasing conditions: V_JG_ = 0, −1, −2 V (V_DS_ = 1 V, V_BG_ = 0 V). Figure 3d presents the corresponding CPD profiles in the source-to-drain direction, as indicated by the dashed arrow in Figure 3c (V_JG_ = 0 V). Note that an increase in CPD is indicative of a higher potential barrier between the source and drain. The highest potential barrier—and the highest CPD—are recorded for V_JG_ = −2 V, as now the depletion areas on both sides of the conducting channel overlap and the channel is practically fully depleted from carriers (no current is measured between the source and drain). Conversely, I_DS_ increases linearly for V_JG_ = 0 V (see ref. [33]), and the conducting channel can be treated as a cone-shaped slab of a doped semiconductor. In this regime, we can use Ohm’s law to extract the diameter of the EFN by using the following known expression: J_DS_ = I_DS_/A = q µ_e_ n E_x_, where J_DS_ is the source-drain current density, A is the diameter of the EFN, q is the elementary charge, µ_e_ is the electron mobility of the EFN, n is the free electron density, and E_x_ is the local electric field. To determine E_x_, we differentiate the CPD along the x-axis: Ex = *d*|CPD|/*dx*). The free carrier density is set equal to the donor concentration (N_D_), which was measured by using TOF-SIMS (N_D_ = 4.0 ± 0.2 × 10^17^ cm^−3^), and the corresponding electron bulk mobility was calculated (µ_e_ = 430 ± 10 cm^2^·V/s). Figure 3e presents the calculated diameter along the source-drain axis of the EFN. Note that the diameter decreases toward the drain due to the potential difference along the EFN, which induces a pinch-off near the drain (in other words, the effective V_JG_ near the drain is higher than the effective V_JG_ near the source due to the potential gradient between the source and drain).

## 5. Gas-Sensing with an EFN Gas Sensor: Sensor Response, Tunable Sensitivity and Dynamic Range, and LOD

For sensing experiments, the EFN is kept in a sealed chamber under a continuous flow of nitrogen, which serves both as the carrier gas and for diluting the target molecules. The sensing experiments take place at room temperature. We define the sensor response as the change in I_DS_ upon the introduction of the target molecule in the following manner: ΔI/I_0_ = (I_0_ − I_f_)/I_0_, where I_0_ = I_DS_ in a nitrogen environment prior to exposure, and I_f_ = I_DS_ after exposing the EFN to the target molecule. The below reviews the basic sensing performance of the EFN gas sensor by using the target molecule ethanol [35] (a performance similar to that of ethanol was also demonstrated for acetone [35]). Ethanol adsorbs on the silanol (Si–OH) groups of SiO_2_ by hydrogen bonding [37,38,39], and it is also likely to adsorb by weakly bonding to the siloxane (Si–O–Si) sites under exposure to a high ethanol concentration [37]. Ethanol adsorption induces a change in the SiO_2_ surface charge density and, therefore, it serves as a molecular gate. The electric field generated by the induced negative surface charge depletes the conducting channel (or EFN) that is sustained in the underlying SOI, which entails a decrease in I_DS_. 

**Figure 4:**
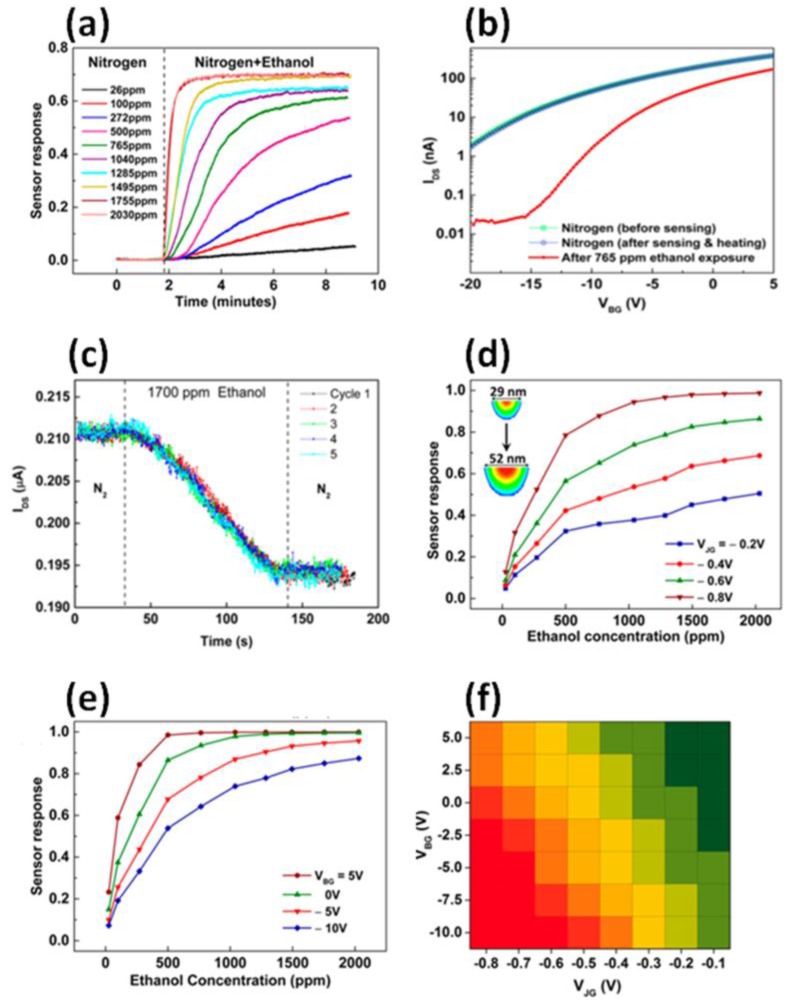
Ethanol sensing with an EFN gas sensor. (**a**) Sensor response vs time for various ethanol concentrations; (**b**) EFN gas sensor recovery using ex situ heating; (**c**) reproducibility measurements of the EFN gas sensor; (**d**) sensor response for various ethanol concentrations at various V_JG_ values; (**e**) sensor response to various ethanol concentrations at various V_BG_ values; (**f**) color map of the sensor response of the EFN gas sensor under different biasing conditions. Reprinted with permission from [35]. Copyright 2016 American Chemical Society.

Figure 4a presents the response of an EFN gas sensor, as function of time, to various concentrations of ethanol as the target molecule (V_JG_ = −0.2 V, V_BG_ = −14 V). The sensor response is proportional to the concentration of the target molecule, such that higher concentrations are positively correlated with the concentration of the target molecule, and for each concentration, the EFN reaches equilibrium after several minutes. In addition, each concentration is characterized by a respective increase in the slope of I_DS_, allowing real-time sensing by monitoring the slope of I_DS_. Figure 4b presents EFN recovery after ethanol adsorption using ex situ heating (60 °C for 4 min), and Figure 4c presents five cycle reproducibility measurements, each exposing the EFN to 1700 ppm of ethanol. Figure 4d,e presents the response of the EFN gas sensor upon exposure to various concentrations of ethanol under various biasing conditions of V_JG_ and a constant V_BG_ = −3 V, and under various biasing conditions of V_BG_ and a constant V_JG_ = −0.8 V, respectively. The sensor response upon V_JG_ and V_BG_ biasing is evident: the greater the negativity of either V_JG_ or V_BG_, the higher the response. Therefore, a small EFN diameter and a shallow EFN (namely, an EFN that is close to the SOI/active oxide interface) are the optimal conditions for enhanced sensor response, as summarized in Figure 4f. It is also evident that the higher the concentration of the target molecule, the higher the sensor response—until a certain concentration is reached, at which the sensor saturates. The sensor response to ethanol saturates at a concentration of 2030 ppm for all biasing conditions. Defining the LOD as the minimum concentration at which the signal-to-noise ratio is higher than or equal to 3, the LOD for ethanol is ~26 ppm for biasing of V_JG_ = −0.8 V at V_BG_ = −12 V. 

The merit of the EFN gas sensor is evident from Figure 4d,e; namely, not only are the size, location, and shape of the channel are tunable, but also the EFN sensing performance is tunable, in accordance with the size of the EFN. As shown in Figure 4d,e, the response of the EFN gas sensor is tunable by properly adjusting the biasing and shape of the EFN—an advantage that is obviously lacking in physical SiNW gas sensors, in which the properties of the channel are set and defined prior to fabrication, and cannot be altered post-fabrication. The tunable EFN sensor response reflects the tunable sensitivity of the sensor, defined as ΔI/(Δmolecule concentration). A higher sensitivity is realized by using a smaller and shallower EFN (negative biasing of V_JG_ and V_BG_), whereas a lower sensitivity is realized by using a wide and “deep” bulky EFN (for example, V_JG_ = V_BG_ = 0 V). The EFN is most sensitive at V_JG_ = −0.8 V (corresponding to an EFN diameter of 29 nm), whereas modifying V_JG_ to −0.2 V (EFN diameter = 52 nm) produces a less sensitive EFN. We reported a 27-fold increase in sensitivity, achieved by reducing the effective diameter of the EFN from 46 nm to 16 nm [33]. 

The dynamic detection range of a gas sensor is the range of concentration of target molecules over which the sensor provides a meaningful response toward a perturbation in target analyte concentration [40]. Typically, the high end of the dynamic range is limited by the saturation effects of the sensor response. The ability to electrostatically manipulate the EFN response toward a given concentration of the target molecule directly leads to the large dynamic range of the sensor [35]. This is evident in Figure 4d; the EFN is most sensitive to low concentrations, with V_JG_ = −0.8 V (diameter of 29 nm), but the EFN response saturates above ~1000 ppm. For V_JG_ = −0.6 V, the EFN is less sensitive but the dynamic range is higher, as the response saturates at ~1500 ppm. Finally, for V_JG_ = −0.2 V (wide channel), the EFN shows the lowest sensitivity, but the dynamic range is greater than the maximum concentration of 2000 ppm shown in the graph. A tunable dynamic range can also be achieved by manipulating V_BG_, as shown in Figure 4e. V_BG_ = −10 V (V_JG_ = −0.7 V) provides a very sensitive EFN gas sensor for low concentrations but saturates at ~500 ppm, whereas V_BG_ = 5 V provides a lower sensitivity but a wide dynamic range (higher than 2000 ppm). Thus, by adequately adjusting the biasing of V_JG_ and V_BG_, both high sensitivity and a high dynamic range can be achieved with a single EFN gas sensor. 

**Figure 5:**
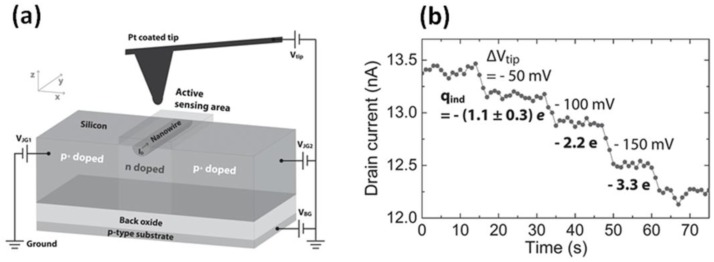
Limit of detection (LOD) of an EFN gas sensor. (**a**) Schematic illustration of the scanning gate microscopy methodology; (**b**) an I_DS_ vs time curve, in which the small steps in I_DS_ are due to the induced atomic force microscopy (AFM) tip charge, which sets the EFN gas sensor electrostatic LOD (eLOD) to the range of a single elementary charge. Reprinted from [41], with permission from Wiley Publishing.

Finally, the LOD of an EFN gas sensor was measured by using scanning gate microscopy (SGM)—a method in which the tip of an atomic force microscope (AFM) is used as a local top gate emulating the induced charge of adsorbed target molecules (Figure 5a) [41]. An analytic electrostatic model was developed to describe the tip-induced charge. This methodology reflects the electrostatic LOD (eLOD), which considers only the charge induced by the target molecule and disregards any interaction between the target molecule and the surface. Figure 5b presents I_DS_ vs time measurements, in which the downward steps in I_DS_ are a response to the application of −50 mV steps at the tip of the AFM (ΔV_tip_). The analytic electrostatic model was applied to the measurements and translated the I_DS_ steps to induced charge; it was shown that the steps in I_DS_ are indicative of an eLOD in the range of a single elementary charge. 

## 6. Conclusions

The EFN gas sensor is a novel gas-sensing platform, which is of low cost, robust, and suitable for mass production. The heart of the EFN technology is the tunable size, shape, and location of the channel. The electrostatic tuning of the size and shape of the EFN elicits a tunable sensitivity and dynamic range. Currently, the main research challenge is to explore and develop an appropriate selectivity scheme for EFN gas sensors to support the realization of an “electronic nose”, based on EFN gas sensors.
